# Association of unsweetened and sweetened tea consumption with the risk of new-onset chronic kidney disease: Findings from UK Biobank and Coronary Artery Risk Development in Young Adults (CARDIA) study

**DOI:** 10.7189/jogh.13.04094

**Published:** 2023-10-20

**Authors:** Mengyi Liu, Yanjun Zhang, Ziliang Ye, Sisi Yang, Yuanyuan Zhang, Panpan He, Chun Zhou, Fan Fan Hou, Xianhui Qin

**Affiliations:** Division of Nephrology, Nanfang Hospital, Southern Medical University; National Clinical Research Centre for Kidney Disease; State Key Laboratory of Organ Failure Research; Guangdong Provincial Institute of Nephrology; Guangdong Provincial Key Laboratory of Renal Failure Research, Guangzhou, China

## Abstract

**Background:**

The association between tea consumption and chronic kidney disease (CKD) remained inconsistent. We aimed to evaluate the association of tea consumption with new-onset CKD and examine the effects of common additives (milk and sweeteners) and genetic variations in caffeine metabolism on the association.

**Methods:**

176 038 and 3104 participants free of CKD at baseline in the United Kingdom Biobank (UK Biobank) and Coronary Artery Risk Development in Young Adults (CARDIA) study were included, respectively. Dietary information was collected using 24-hour dietary recall questionnaires. The study outcome was new-onset CKD.

**Results:**

In the UK Biobank, during a median follow-up of 12.13 years, 3535 (2.01%) participants developed CKD. Compared with tea non-consumers, the risk of new-onset CKD was significantly lower in unsweetened tea consumers (hazard ratio (HR) = 0.84, 95% confidence interval (CI) = 0.76-0.93), but not in sweetened tea consumers (HR = 0.96, 95% CI = 0.85-1.08), regardless of whether milk was added to tea. Accordingly, relative to tea non-consumers, the adjusted HRs (95% CIs) of new-onset CKD for participants who reported drinking unsweetened tea 1.5 or fewer, >1.5 to 2.5, >2.5 to 3.5, >3.5 to 4.5, and >4.5 drinks/d were HR = 0.86, 95% CI = 0.75-0.99; HR = 0.88, 95% CI = 0.78-1.00; HR = 0.83, 95% CI = 0.73-0.94; HR = 0.83, 95% CI = 0.72-0.95; and HR = 0.86, 95% CI = 0.75-0.99. Moreover, the association of unsweetened tea consumption with new-onset CKD was stronger among those with faster genetically predicted caffeine metabolism levels, although the interaction was insignificant (*P*-value interaction = 0.768). Consistently, in the CARDIA study, compared with tea non-consumers, a significantly lower risk of new-onset CKD was found in unsweetened tea consumers (HR = 0.80, 95% CI = 0.65-0.98) but not in sweetened tea consumers (HR = 0.97, 95% CI = 0.70-1.34).

**Conclusions:**

Compared with tea non-consumers, consumption of unsweetened tea, but not sweetened tea, was significantly associated with a lower risk of new-onset CKD, regardless of whether milk was added.

Chronic kidney disease (CKD) is a global health and socioeconomic burden, affecting 697 million worldwide [1]. CKD is associated with poor health outcomes, including renal failure, cardiovascular disease (CVD), and all-cause mortality [2,3]. Therefore, it is important to identify more modifiable risk factors for CKD at an early stage to establish primary preventive measures. Tea is among the most frequently consumed beverages in the United Kingdom (UK) and worldwide [4,5]. As an extract made from the leaves of the plant *Camellia sinensis*, tea contains various kinds of biomolecules, such as catechins, oligomeric flavonoids, and flavonols, which have been shown to possess antioxidative and anti-inflammatory properties [6,7]. Accordingly, tea consumption has been reported to be related to risk reduction of multiple health outcomes, such as obesity, hypertension, and type two diabetes [8,9], all of which are major causes of CKD. Nevertheless, studies examining the associations between tea consumption and renal outcomes have yielded mixed results. While some previous studies failed to observe any relationship between tea consumption and the risk of incident CKD [10] or end-stage renal disease (ESRD) [11] or annual changes in estimated glomerular filtration rate (eGFR) [12] in the general population, another study showed that an increase in tea consumption was related to a lower likelihood of CKD and increased levels of eGFR [13]. This discrepancy may be due to the relatively small sample sizes of previous studies [10-13], failure to consider the effects of milk or sweetener additives on tea, and different definitions of the reference group and renal outcomes. Black tea was especially commonly consumed with milk or sweeteners in Western countries [8]. Considering the potential positive association between the consumption of sugar-sweetened beverages (SSBs) and CKD [14], it is necessary to assess the effect of sweetened and unsweetened tea on CKD. In addition, tea is a major contributor to dietary caffeine, though it is usually less caffeinated than coffee [15]. Given the growing concerns regarding high caffeine intake among individuals with slow caffeine metabolism [16-18], exploring the modifying effects of genetic variation in caffeine metabolism on the relation of tea consumption with CKD may yield new insights for clinical and public health.

To address the above knowledge gaps, we aimed to evaluate the dose-response association of tea consumption with new-onset CKD and examine the effects of common additives (milk and sweeteners) and genetic variations in caffeine metabolism on the association.

## METHODS

### Data source and study population

The UK Biobank is a large prospective, observational study designed to examine the role of comprehensive exposures in health and diseases. As previously described [19,20], between 2006 and 2010, the UK Biobank enrolled approximately 500 000 adult volunteer participants aged 37-73 years from one of 22 assessment centres across the UK (England, Wales and Scotland). Extensive data were collected through questionnaires on sociodemographic, lifestyle, psychosocial factors, and medical history. Data collection comprised touchscreen questionnaires and verbal interviews by a trained staff member. Participants also underwent a standardised portfolio of physical measurements and provided blood, urine, and saliva samples. The UK Biobank was approved by the North West Research Ethics Committee, and all participants signed informed consent.

The current analysis was restricted to participants who completed at least one online 24-hour dietary recall questionnaire and had complete information on kidney function. Participants with prior CKD (self-reported history of CKD or CKD diagnosis before the date of baseline assessment; or an eGFR<60 mL (mL)/min (min)/1.73 m^2^ (m^2^); or the presence of urine albumin: creatinine ratio (UACR)≥30 mg (mg)/gram (g)); or implausible total energy intake (male with <800 kilocalories (kcal)/d (d) or >4200 kcal/d or female with <600 kcal/d or >3500 kcal/d); or inconsistent addition status of sweeteners in any dietary recall were further excluded. Therefore, 176 038 participants were enrolled in the present analyses (Figure S1 in the **Online Supplementary Document**).

### Dietary assessments

Dietary information was collected using a web-based 24-hour dietary recall questionnaire called the Oxford WebQ, which was used to investigate the types and quantities of foods consumed, including beverages and daily nutrient intake, during the previous 24 hours and has been validated in detail elsewhere [21]. Participants were invited to complete the questionnaire on five occasions over one year to account for seasonal variations in dietary intake between April 2009 and June 2012.

In each 24-hour dietary recall, participants indicated how many drinks of tea and coffee they had consumed. If participants drank tea or coffee, they would be asked about the types of tea or coffee they usually drank and whether they added milk or sweeteners (including sugar and artificial sweeteners). The online diet questionnaire provided some specifications for regular drinks (e.g. mug or cup) to reduce confusion about size. Because participants could complete the 24-hour dietary recall up to five times, we classified a participant drinking tea at any one dietary recall as a tea consumer; all others were classified as non-consumers. For tea consumers, the average number of drinks across multiple dietary recalls was calculated as a marker of habitual intake.

### Covariates measurements

We selected covariates a priori based on biological plausibility and prior empirical evidence associated with tea consumption and kidney function. Detailed information on covariates was available through several touchscreen computer-based questionnaires and a face-to-face interview with a trained researcher. We collected data that included basic demographics (sex and age), ethnicities, educational attainment, and behavioural factors (smoking status, alcohol consumption, moderate and vigorous physical activity, and dietary consumption). Optimal physical activity was defined as more than four days of vigorous/moderate physical activity in a typical week. Body mass index (BMI) was constructed from height and weight measured during the initial assessment centre visit. We measured blood pressure (BP) twice manually (manual sphygmomanometer) or automatically (Omron HEM-7015IT digital blood pressure monitor), and the mean value of the two measurements was used to minimise the measurement error. Procedures for collecting and processing baseline blood and urine samples have been previously described and validated [22]. We performed the biochemistry measures at a dedicated central laboratory, including creatinine, lipids (triglycerides (TG)), total cholesterol (TC), high-density lipoprotein cholesterol (HDL-C)), and UACR.

### Genetic caffeine metabolism score

Detailed information about genotyping and quality control in the UK Biobank study has been described previously [22]. By using four common single-nucleotide polymorphisms (rs2472297, rs56113850, rs6968554, and rs17685) that are associated with blood caffeine metabolite levels and located in or near genes involved in caffeine metabolism, we derived the weighted genetic caffeine metabolism score (wCMSG4) by summing the number of alleles multiplied by their beta (β)-coefficients [23]. We created three categories for wCMSG4 based on the tertiles of wCMSG4, and participants with higher scores were predicted to have faster caffeine metabolism.

### Ascertainment of outcome

The study outcome was new-onset CKD, based on medical history and linked to hospital admissions and death registry records data. The website of the UK National Health Service (NHS) (http://digital.nhs.uk/services) showed the linkage procedure in detail. CKD was defined according to the International Classification of Diseases (ICD) code and the Office of Population Censuses and Surveys Classification of Interventions and Procedures (OPCS) code (Table S1 in the **Online Supplementary Document**). We calculated the follow-up person-time for each participant from the date of first assessment until the date of death, first date of outcome diagnosis, date of loss to follow-up, or end of follow-up, whichever came first.

### Statistical analysis

We presented population characteristics as mean (standard deviation (SD)) for continuous variables and proportions for categorical variables. We performed comparisons of characteristics according to tea consumption (tea non-consumers, unsweetened tea consumers, and sweetened tea consumers) by χ^2^ tests for categorical variables and one-way analysis of variance for continuous variables.

We used restricted cubic spline Cox regression to explore the shape of the dose-response relation between tea consumption and new-onset CKD. The consumption was then categorised into the following five groups: greater than zero to 1.5 drinks/d, greater than 1.5 to 2.5 drinks/d, greater than 2.5 to 3.5 drinks/d, greater than 3.5 to 4.5 drinks/d, and greater than 4.5 drinks/d, which is equivalent to an average of one, two, three, four, and five or more drinks per day. Cox proportional hazard models were used to estimate the hazard ratio (HR) and 95% confidence interval (CI) of new-onset CKD for tea consumption with non-consumers as the referent group. In the multivariable models, we adjusted potential confounders that were known to be traditional or suspected risk factors for CKD, including sex, age, ethnicity, educational attainment, smoking status, alcohol consumption, physical activity, total energy intake, coffee consumption, BMI, systolic BP (SBP), diastolic BP (DBP), glucose, TG, TC, HDL-C, eGFR, and UACR. Percentages of missing values of covariates were less than 1.0% except for physical activity (3.0%), glucose (8.7%) and HDL-C (8.7%). We coded missing data as a missing indicator category for categorical variables and with mean values for continuous variables.

To validate the result, we further examined the associations of tea consumption and new-onset CKD in an independent, prospective cohort: Coronary Artery Risk Development in Young Adults (CARDIA) study, which has repeatedly comprehensive data collection on tea consumption and renal function. The CARDIA study is an ongoing longitudinal study that examined the development and determinants of clinical and subclinical cardiovascular disease and their risk factors. It began in 1985 and 1986 (baseline) with a group of 5115 males and females aged 18-30 years. The CARDIA study defined new-onset CKD as an eGFR<60 mL/min/1.73 m^2^ or UACR≥30 mg/g. More detailed information on the methods regarding validation analysis in CARDIA was provided in the Methods Section in the **Online Supplementary Document**.

To evaluate the potential modification effects of caffeine, we performed stratified analyses according to genetically predicted caffeine metabolism (quartiles of wCMSG4). As additional exploratory analyses, possible modifications were also assessed for variables including age (<60 or ≥60 years), sex (male or female), BMI (<30 or ≥30 kgmes (kg)/m^2^), smoking status (never or ever), alcohol consumption (<1 or ≥1 time/week), optimal physical activity (yes or no), and energy intake (<median or ≥ median). We assessed the modifying effects by modelling the cross-product term of the stratifying variable with tea consumption.

We performed a series of sensitivity analyses to assess the robustness of the results. First, we excluded participants with new-onset CKD during the first two years of follow-up. Second, we excluded participants with missing covariates. Third, consumption of water, low/non-sugar SSBs, and SSBs were further adjusted. Fourth, we explored the relationship between tea consumption and new-onset CKD, considering the addition of sugar and artificial sweeteners separately. A two-tailed *P*-value <0.05 was considered statistically significant in all analyses. Analyses were performed using software R (Version 4.3.0) (R Core Team, Vienna, Austria).).

## RESULTS

### Study participants and baseline characteristics

Of the 176 038 participants in the current study, 96 269 (54.7%) were females, with a mean age = 55.91 (SD = 7.92) years. Approximately 83.45% of the participants reported drinking tea, and 80.54% of the tea consumers added sweeteners. Compared with tea non-consumers, tea consumers were older and tended to have higher energy intake and physical activity levels, while lower levels of coffee consumption and BMI (**Table 1**). Unsweetened tea consumers tended to be females, White, and non-smokers and were more likely to have higher levels of education, alcohol consumption, HDL-C, and UACR but lower levels of BP and TG. Conversely, sweetened tea consumers tended to be males, non-White, and smokers and were more likely to have lower levels of education, alcohol consumption, and TC but higher BP.

**Table 1:** Baseline population characteristics according to tea consumption categories in the UK Biobank

Characteristics	Non-consumers	Tea consumers	
		**Unsweetened**	**Sweetened**
**Number of participants**	29 127	118 319	28 592
Age (years)*	54.80 (8.15)	56.15 (7.78)	56.07 (8.16)
**Male†**	14 175 (48.67)	49 053 (41.46)	16 541 (57.85)
**White†**	27 777 (95.37)	115 109 (97.29)	26 352 (92.17)
**Education†**			
No secondary education	2722 (9.39)	8217 (6.97)	3564 (12.55)
Secondary education	14 948 (51.54)	54 307 (46.06)	16 011 (56.39)
University degree	11 330 (39.07)	55 387 (46.97)	8818 (31.06)
**Smoking status†**			
Never	15 822 (54.43)	70 347 (59.58)	13 798 (48.4)
Former	9992 (34.37)	41 146 (34.85)	10 919 (38.3)
Current	3254 (11.19)	6587 (5.58)	3790 (13.29)
**Alcohol consumption (times/week)†**			
<1	8924 (30.65)	28 440 (24.04)	9214 (32.24)
1-2	6772 (23.26)	30 208 (25.54)	7022 (24.57)
3-4	6536 (22.45)	32 330 (27.33)	6038 (21.13)
>4	6881 (23.64)	27 308 (23.09)	6301 (22.05)
Physical activity (days per week)*			
Moderate	3.44 (2.34)	3.57 (2.27)	3.54 (2.31)
Vigorous	1.83 (1.94)	1.87 (1.85)	1.78 (1.92)
Energy intake (kilocalorie per day)*	2011.6 (581.40)	2037.43 (530.59)	2144.75 (591.31)
Coffee consumption (number of drinks per day)*	2.88 (1.79)	1.53 (1.34)	1.34 (1.39)
Biological factors*			
Body mass index, kilogram per square meter	27.82 (5.02)	26.55 (4.4)	27.13 (4.42)
Systolic blood pressure, millimetres of mercury	136.33 (17.78)	135.93 (18.00)	137.52 (17.83)
Diastolic blood pressure, millimetres of mercury	82.25 (10.02)	81.36 (9.84)	82.39 (9.98)
Glucose, millimoles per litre	5.13 (1.22)	5.06 (1.01)	5.08 (0.99)
Triglycerides, millimoles per litre	1.77 (1.06)	1.61 (0.93)	1.83 (1.06)
Total cholesterol, millimoles per litre	5.71 (1.14)	5.74 (1.11)	5.64 (1.12)
High-density lipoprotein cholesterol, millimoles per litre	1.43 (0.38)	1.51 (0.39)	1.39 (0.35)
Estimated glomerular filtration rate, millilitres per minute per 1.73 m^2^	93.39 (11.94)	91.72 (11.60)	91.01 (12.23)
Urine albumin-to-creatinine ratio, milligram per gram	8.36 (5.48)	9.48 (5.78)	8.37 (5.44)

*Values are presented as means (standard deviation).

†Values are presented as N (sample) and percentages.

### Association between tea consumption and new-onset CKD

**Figure 1:**
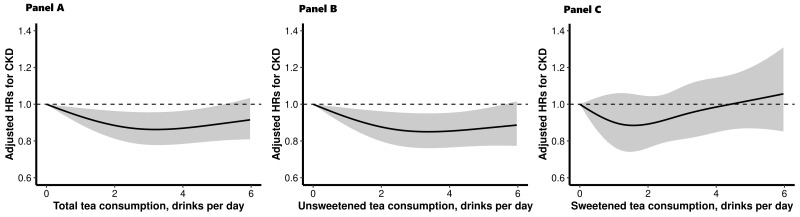
Dose-response association of total, unsweetened, and sweetened tea consumption and new-onset CKD. **Panel A.** Dose-response association of total tea consumption and new-onset CKD. **Panel B.** Dose-response association of unsweetened tea consumption and new-onset CKD. **Panel C.** Dose-response association of sweetened tea consumption and new-onset CKD. Results were adjusted for age, sex, ethnicities, educational attainment, smoking status, alcohol consumption, physical activity, total energy intake, coffee consumption, body mass index, systolic blood pressure, diastolic blood pressure, glucose, triglycerides, total cholesterol, high-density lipoprotein cholesterol, estimated glomerular filtration rate and urine albumin-to-creatinine ratio. CKD – chronic kidney disease, HR – hazard ratio

During a median follow-up of 12.13 years, 3 535 (2.01%) new-onset cases of CKD were documented. Overall, there was a L-shaped association between tea consumption and the risk of new-onset CKD (**Figure 1**, panel A and Table S2 in the **Online Supplementary Document**). Considering the addition of sweeteners in tea, there was an L-shaped association between unsweetened tea consumption and new-onset CKD (**Figure 1**, panel B), while a null association between sweetened tea consumption and new-onset CKD (**Figure 1**, panel C). Relative to tea non-consumers, adjusted HRs (95% CIs) of new-onset CKD for participants who reported drinking unsweetened tea 1.5 or fewer, >1.5 to 2.5, >2.5 to 3.5, >3.5 to 4.5, and >4.5 drinks/d were HR = 0.86, 95% CI = 0.75-0.99; HR = 0.88, 95% CI = 0.78-1.00; HR = 0.83, 95% CI = 0.73-0.94; HR = 0.83, 95% CI = 0.72-0.95, and HR = 0.86, 95% CI = 0.75-0.99 (**Table 2**). Accordingly, compared with tea non-consumers, a significantly lower risk of new-onset CKD was found in unsweetened tea consumers (HR = 0.84, 95% CI = 0.76-0.93), but not in sweetened tea consumers (HR = 0.96, 95% CI = 0.85-1.08) (**Table 3**).

**Table 2:** Hazard ratio (95% confidence interval) of new-onset chronic kidney disease according to unsweetened and sweetened tea consumption in the UK Biobank

Tea additives	Tea non-consumers	Tea consumers (drinks per day)				
		**0.5-≤1.5**	**>1.5-2.5**	**>2.5-3.5**	**>3.5-4.5**	**>4.5**
**Unsweetened tea**						
Events (rates*)	606 (1.7)	327 (1.3)	461 (1.5)	474 (1.5)	409 (1.5)	488 (1.7)
Adjusted model 1†	Reference	HR = 0.72 (95% CI = 0.63-0.83)	HR = 0.76 (95% CI = 0.67-0.86)	HR = 0.75 (95% CI = 0.67-0.85)	HR = 0.79 (95% CI = 0.70-0.89)	HR = 0.89 (95% CI = 0.79-1.01)
Adjusted model 2†	Reference	HR = 0.86 (95% CI = 0.75-0.99)	HR = 0.88 (95% CI = 0.78-1.00)	HR = 0.83 (95% CI = 0.73-0.94)	HR = 0.83 (95% CI = 0.72-0.95)	HR = 0.86 (95% CI = 0.75-0.99)
**Sweetened tea**						
Events (rates*)	606 (1.7)	119 (1.8)	153 (2.0)	168 (2.3)	148 (2.5)	182 (2.8)
Adjusted model 1†	Reference	HR = 0.96 (95% CI = 0.79-1.17)	HR = 1.01 (95% CI = 0.85-1.21)	HR = 1.11 (95% CI = 0.94-1.32)	HR = 1.22 (95% CI = 1.02-1.46)	HR = 1.37 (95% CI = 1.16-1.62)
Adjusted model 2†	Reference	HR = 0.92 (95% CI = 0.75-1.12)	HR = 0.93 (95% CI = 0.77-1.11)	HR = 0.96 (95% CI = 0.80-1.15)	HR = 0.98 (95% CI = 0.80-1.20)	HR = 1.01 (95% CI = 0.83-1.23)

*Incidence rates were presented as per 1000 person years.

**†**Model 1: adjusted for age, sex, and ethnicities. Model 2: adjusted for the covariates in Model 1 and further adjusted for educational attainment, smoking status, alcohol consumption, physical activity, total energy intake, coffee consumption, body mass index, systolic blood pressure, diastolic blood pressure, glucose, triglycerides, total cholesterol, high-density lipoprotein cholesterol, estimated glomerular filtration rate and urine albumin-to-creatinine ratio.

**Table 3:** Hazard ratio (95% confidence interval) of new-onset chronic kidney disease among unsweetened and sweetened tea consumers in the UK Biobank and Coronary Artery Risk Development in Young Adults (CARDIA) study

Study	Tea non-consumers	Tea consumers	
		**Unsweetened**	**Sweetened**
**UK Biobank**			
N	29 127	118 319	28 592
Events	606	2159	770
Incidence rates*	1.7	1.5	2.3
Adjusted model 1†	Reference	HR = 0.78 (95% CI = 0.71-0.85)	HR = 1.12 (95% C = 1.00-1.24)
Adjusted model 2†	Reference	HR = 0.84 (95% CI = 0.76-0.93)	HR = 0.96 (95% CI = 0.85-1.08)
**CARDIA study**			
N	871	1972	261
Events	163	272	49
Incidence rates*	7.4	5.2	7.2
Adjusted model 1†	Reference	HR = 0.72 (95% CI = 0.59-0.88)	HR = 0.94 (95% CI = 0.68-1.29)
Adjusted model 2†	Reference	HR = 0.80 (95% CI = 0.65-0.98)	HR = 0.97 (95% CI = 0.70-1.34)

*Incidence rates were presented as per 1000 person years.

**†**Model 1: Adjusted for age, sex, and ethnicities; model 2: adjusted for the covariates in model 1 and further adjusted for educational attainment, smoking status, alcohol consumption, physical activity, total energy intake, coffee consumption, body mass index, systolic blood pressure, diastolic blood pressure, glucose, triglycerides, total cholesterol, high-density lipoprotein cholesterol, estimated glomerular filtration rate and urine albumin-to-creatinine ratio (only for the UK Biobank).

When further considering the addition of milk, we observed similar results for unsweetened tea consumption (including tea not adding milk or sweeteners and tea adding milk only) and sweetened tea consumption (including tea adding sweeteners only and tea adding both milk and sweeteners), regardless of whether milk was added to tea (Table S3 in the **Online Supplementary Document**).

In the sensitivity analyses, we observed consistent trends for unsweetened and sweetened tea consumption when further excluding participants who had an outcome event during the first two years of follow-up, or excluding those with missing covariates, or further adjustments for consumption of water, low/non-sugar SSBs, and SSBs (Table S4 in the **Online Supplementary Document**). In addition, there was no association between sweetened tea consumption and new-onset CKD whether sugar or artificial sweeteners were added to tea (Table S4 in the **Online Supplementary Document**).

### Validation analysis in the CARDIA study

In the CARDIA study, 3104 participants were included in the final analysis (Figure S2 in the **Online Supplementary Document**); 1817 (58.54%) were females, with a mean age = 25.20 (SD = 3.60). Approximately 71.94% of the participants reported drinking tea, of which 88.31% and 11.69% were unsweetened and sweetened tea consumers. Detailed characteristics are shown in Table S5 in the **Online Supplementary Document**.

During a median follow-up of 29.70 years, 484 (15.59%) participants occurred new-onset CKD. Compared with tea non-consumers, a significantly lower risk of new-onset CKD was found in unsweetened tea consumers (HR = 0.80, 95% CI = 0.65-0.98) but not in sweetened tea consumers (HR = 0.97, 95% CI = 0.70-1.34) (**Table 3**).

### Stratified analyses by potential effect modifiers

**Figure 2:**
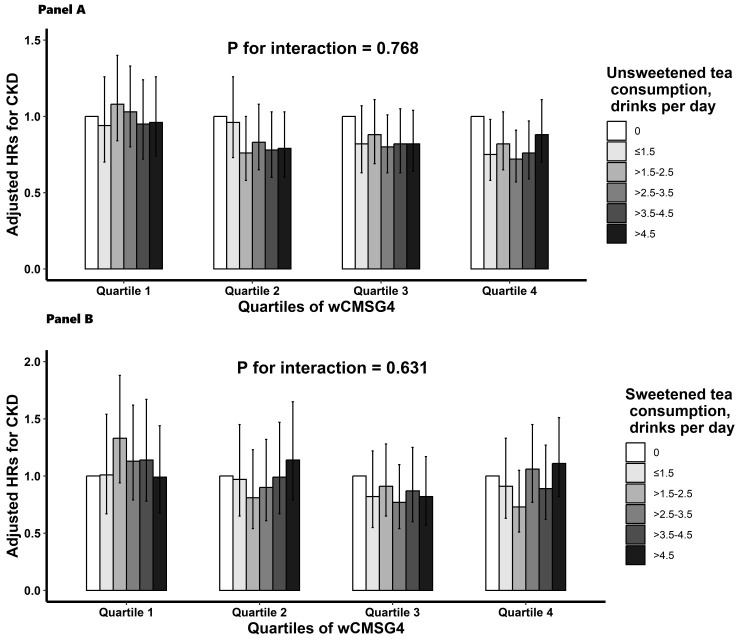
Association of tea consumption and new-onset CKD stratified by tertiles of the wCMSG4 levels. **Panel A.** Association of unsweetened tea consumption and new-onset CKD stratified by tertiles of the wCMSG4 levels. **Panel B.** Association of sweetened tea consumption and new-onset CKD stratified by tertiles of the wCMSG4 levels. Results were adjusted for age, sex, ethnicities, educational attainment, smoking status, alcohol consumption, physical activity, total energy intake, coffee consumption, body mass index, systolic blood pressure, diastolic blood pressure, glucose, triglycerides, total cholesterol, high-density lipoprotein cholesterol, estimated glomerular filtration rate and urine albumin-to-creatinine ratio. CKD – chronic kidney disease, HR – hazard ratio, wCMSG4 – weighted genetic caffeine metabolism scores

We performed stratified analyses to assess the relation between tea consumption and the risk of new-onset CKD in different wCMSG4 (quartiles) categories (**Figure 2**). Although wCMSG4 did not significantly modify the association of unsweetened tea (*P*-value interaction = 0.768) (**Figure 2**, panel A) and sweetened tea (*P*-value interaction = 0.631) (**Figure 2**, panel B) consumption with the risk of new-onset CKD, we observed more obvious inverse association between unsweetened tea consumption and the risk of new-onset CKD in those with higher wCMSG4 (the 2^nd^, 3^rd^, and 4^th^ quartile).

In the additional exploratory analyses, none of the other variables, including age, sex, BMI, smoking status, alcohol consumption, optimal physical activity, and energy intake, showed significant effect modification on the relation of unsweetened tea consumption and the risk of new-onset CKD (all *P* for interaction >0.05) (Table S6 in the **Online Supplementary Document**).

## DISCUSSION

In this large-scale, population-based prospective study, we observed an inverse association between unsweetened tea consumption and the risk of new-onset CKD, but not for sweetened tea consumption, regardless of whether milk was added to the tea. The results were more obvious for unsweetened tea in those with faster genetically predicted caffeine metabolism. Meanwhile, a validation analysis in the CARDIA study consistently found a significantly lower risk of new-onset CKD associated with unsweetened tea but not sweetened tea.

Before our study, a few studies investigated the association between tea consumption and CKD but reported inconsistent results. A recent Mendelian randomisation study using summary-level data from the UK Biobank and the Chronic Kidney Disease genetics (CKDGen) consortium showed that an increase in tea consumption appeared to be related to the reduced risk of CKD and an increase in eGFR [13]. Nevertheless, in the Doetinchem Cohort Study, including 4722 Dutch adults aged 26-65 years, tea consumption was not significantly associated with subsequent annual changes in eGFR [12]. Of note, the study outcome, changes in GFR, could not indeed reflect the abnormal GFR loss that would persist and eventually lead to stage three CKD over time. The Singapore Chinese Health Study, a prospective cohort study of 63 257 participants aged 45-74 years, found that neither black tea intake nor green tea intake was associated with the risk of ESRD over an average follow-up of 17 years [11]. However, the study did not control the effect of total tea intake. For example, when assessing the effect of black tea intake, green tea drinkers were included in the reference group, which might attenuate the findings toward the null under the assumption that tea intake was beneficial for renal health. In addition, in the Tehran Lipid and Glucose Study, which included 1780 adult Iranians, tertile categories of tea intakes were associated with a lower risk of CKD (HR = 0.89-0.92) during a median follow-up of six years [10], although the results were not statistically significant possibly due to insufficient statistical power. Overall, although milk or sweeteners are commonly added to tea in Western countries [8], which may mask or counteract the benefits of tea consumption, none of the previous studies have assessed the effects of tea additives (milk and/or sweeteners), and thus could not accurately measure the association between tea intake and renal health. Thus, the relationship of tea intake with new-onset CKD remains unclear.

Using a prospective cohort design with a large sample size, a high proportion of tea consumption, a wide range of tea intake, and detailed information on tea additives (milk and/or sweeteners), our study first demonstrated a significant inverse relationship between unsweetened tea consumption and the risk of new-onset CKD, while sweetened tea intake was not associated with the risk of new-onset CKD. In addition, there was a similar, or even more pronounced, inverse association between unsweetened tea consumption and the risk of CKD among participants who added milk to their tea compared to those who did not add milk to their tea, suggesting that sweeteners should be used with caution, and that unsweetened tea with added milk may be a healthier option.

While biological mechanisms underlying our observed inverse association between unsweetened tea intake and new-onset CKD remain to be determined, our findings are biologically plausible. First, kidneys are vulnerable to redox imbalances and oxidative stress (OS), and thus, OS could result in alteration in renal blood flow, sodium/fluid retention, inflammation and fibrotic changes in the kidney, and onset of proteinuria [24]. Tea and its bioactive components, such as polyphenols, polysaccharides, and pigments, are thought to display antioxidant activity through scavenging free radicals, depleting reactive oxygen species (ROS), increasing antioxidant contents, and enhancing antioxidant enzyme activities, thereby protecting kidney function [25,26]. Second, inflammation is another key patho-mechanisms of kidney dysfunction [27]. Tea extracts and their bioactive components may reduce the risk of CKD due to their anti-inflammatory activity, including the regulation of pro-inflammatory and anti-inflammatory factors, as well as the related signalling pathways [26]. Third, a recent study in the UK Biobank found that higher tea consumption (black and green tea) was associated with more favourable levels of cardiometabolic biomarkers, including lower TC and low-density lipoprotein cholesterol, apolipoprotein B and fasting TG, but higher levels of HDL-C [28]. Considering that abnormalities of lipid metabolism have been identified as a possible cause of CKD [29], it is speculated that tea consumption may protect kidney function in part by improving lipid metabolism. However, other mechanisms may also be involved, and future studies are needed to further explore the roles of tea consumption in renal health.

Of note, a previous study has reported that the interaction between tea polyphenols and glucose complexes may lower the antioxidative capacity of tea [30], and thus, it was not surprising that we observed a null association between sweetened tea intake and new-onset CKD. Furthermore, sweeteners per se may contribute to both glomerular and tubular injury by inducing alterations in glomerular pressure and vasopressin, increasing the production of free radicals, and altering the oxidant/antioxidant balance [31-33]. Therefore, it was speculated that the potential dangers of sweeteners may counteract the renal benefit of tea. However, further research is needed to examine the potential mechanisms.

Another point worthy of mention in this study was the weaker association between unsweetened tea consumption and the risk of CKD among individuals with slow caffeine metabolism, although the interaction did not reach statistical significance. Indeed, caffeine has been shown to increase blood pressure and exacerbate renal damage in animal models [34]. It is possible that caffeine, by blocking the A1 and A2A adenosine receptors, while increasing intrarenal angiotensin II levels and the activity of the renal renin-angiotensin system, may permit a greater portion of the systemic blood pressure to be transmitted to glomeruli, thus causing renal injury [34]. As such, we hypothesised that the benefits of tea may be offset by the detrimental effects of caffeine when caffeine metabolism is impaired.

The current study has several potential limitations. First, given the inherent limitations of observational design, the possibility of residual confounding or bias due to unmeasured or unknown factors cannot be ruled out. For example, dietary or medical factors that also contribute to the lower incidence of CKD in unsweetened tea drinkers may not have been included in the analysis. Second, the participants in our study were predominantly of European descent and healthier than the UK general population [35], which may limit generalisability of the findings to other populations. Nevertheless, valid assessments of exposure-disease relationships usually do not require a representative population [35]. Third, data regarding tea consumption was assessed by questionnaires. Therefore, recall bias is possible. However, among the subset of participants who completed the Food Frequency Questionnaire (FFQ) and at least two 24-hour diet recall questionnaires, there was a good correlation between tea intake from the FFQ and mean dietary-recall intakes (correlation coefficient (r) = 0.81) [28]. Fourth, the UK Biobank study used register ICD codes to detect the outcomes, which may have led to the omission of mild CKD, thereby underestimating the true association between unsweetened tea consumption and the risk of CKD. However, in the validation analysis in the CARDIA study with repeat assessments of eGFR and ACR, we observed a similar inverse association of unsweetened tea consumption with the risk of new-onset CKD, defined as an eGFR<60 mL/min/1.73 m^2^ or ACR≥30 mg/g during the follow-up, which further confirmed the beneficial effect of unsweetened tea consumption on renal health. Overall, further studies are necessary to confirm our findings.

## CONCLUSIONS

In conclusion, this large-scale prospective study showed that regardless of whether milk was added, consumption of unsweetened tea, but not sweetened tea, was significantly associated with a lower risk of new-onset CKD. The inverse association was weaker among individuals with slow caffeine metabolism. If further confirmed, our data provide some guidance to tea drinkers and suggest that consumption of unsweetened tea, regardless of whether milk was added to the tea, can be a part of a healthy diet for renal health.

## Additional material


Online Supplementary Document
