# Supplementary material for: Association of unsweetened and sweetened tea consumption with the risk of new-onset chronic kidney disease: Findings from UK Biobank and Coronary Artery Risk Development in Young Adults (CARDIA) study
Source: J Glob Health. 2023 Oct 20;13:04094. doi: 10.7189/jogh.13.04094 (PMC10586794; doi:10.7189/jogh.13.04094)
Supplement: Online Supplementary Document [file jogh-13-04094-s001.pdf]

Supplemental materials

Methods Section: Associations of tea consumption with risk of new-onset CKD in an independent, prospective cohort: Coronary Artery Risk Development in Young Adults (CARDIA) study.

Population and study design

The CARDIA study is an ongoing longitudinal study that examined the development and determinants of clinical and subclinical cardiovascular disease and their risk factors. It began in 1985–1986 (baseline) with a group of 5115 black and white males and females aged 18-30 years. The participants were selected so that there would be balanced proportions in subgroups of race, sex, education and age in each of 4 centers (Birmingham, AL; Chicago, IL; Minneapolis, MN; and Oakland, CA). These same participants were asked to participate in follow-up examinations during 1987–1988 (Y2), 1990–1991 (Y5), 1992–1993 (Y7), 1995–1996 (Y10), 2000–2001 (Y15), 2005–2006 (Y20), 2010–2011 (Y25), and 2015–2016 (Y30) with 90%, 86%, 81%, 77%, 74%, 72%, 72% and 71% retention, respectively. Further details about the study design have been previously published (1). The study was approved by the institutional review boards at each site, and all participants provided written informed consent at each examination.

Two participants who had sex change operations during the course of the study and one participant who dropped out after recruitment were deleted from all datasets. In the current study, we excluded participants without complete information on tea consumption and kidney function (n=714), those with CKD at baseline (n=33), or those who reported an extreme total energy intake (>4200 or <800 kcal/day for males; >3500 or <600 kcal/day for females) (n=698). In order to assess the

independent effect of unsweetened tea and sweetened tea, those drinking both unsweetened tea and sweetened tea (n=563) were further excluded. Finally, a total of 3104 participants were included in the final analysis.

Ascertainment of tea intake

Dietary intake information was collected at baseline, Y7 and Y20 through a validated interviewer-administered CARDIA Diet History Questionnaire (2). Briefly, participants were asked open-ended questions about their eating habits over the past month, including frequency, amount of food consumption, and preparation method.

To reduce measurement errors caused by within-person variation and to best represent the long-term dietary intakes, all values of nutrients in the analyses, if not specified, were presented as the cumulative averages in the main analysis. For example, if new cases of CKD or last follow-up were identified at Y10, Y15 or Y20, the average tea intake from the measurements at baseline and Y7 was used, otherwise, the average tea intake from the measurements at baseline, Y7 and Y20 was used.

Ascertainment of CKD

Serum creatinine concentration was assayed with the Jaffe method at baseline, Y10, Y15, Y20, Y25, and Y30, and the estimated glomerular filtration rate (eGFR) was calculated from the serum creatinine based on the Chronic Kidney Disease Epidemiology Collaboration equation (3). Albuminuria was determined from a single, untimed (spot) urine sample collected at Y10, Y15, Y20, Y25 and Y30. Urinary albumin was measured using nephelometry with a specific anti-albumin monoclonal antibody, and urinary creatinine was measured using the Jaffe method at Y10, Y15 and Y20, and Roche enzymatic method at Y25 and Y30. Urinary Albumin to creatinine ratio (ACR) was calculated as the ratio of urinary albumin (mg) to urinary creatinine (g), and albuminuria was defined as an ACR value ≥30 mg/g.

The primary outcome was new-onset CKD, defined as an eGFR <60 mL/min per 1.73 m^2 or ACR ≥30 mg/g.

Statistical Analysis

We performed analyses in consistency with those in UK Biobank. Cox proportional hazards regression models were used to examine the association between tea intake and new-onset CKD. Models were adjusted for age, sex, ethnicities, educational attainment, smoking status, alcohol consumption, physical activity, total energy intake, coffee consumption, body mass index, systolic blood pressure, diastolic blood pressure, glucose, triglycerides, total cholesterol, high-density lipoprotein cholesterol, and estimated glomerular filtration rate.

References

1. Friedman GD, Cutter GR, Donahue RP, Hughes GH, Hulley SB, Jacobs DR Jr, Liu K, Savage PJ. CARDIA: study design, recruitment, and some characteristics of the examined subjects. *J Clin Epidemiol*. 1988;41(11):1105-16.

2. McDonald A, Van Horn L, Slattery M, Hilner J, Bragg C, Caan B, Jacobs D Jr, Liu K, Hubert H, Gernhofer N, Betz E, Havlik D. The CARDIA dietary history: development, implementation, and evaluation. *J Am Diet Assoc.* 1991;91(9):1104-12.

3. Levey AS, Stevens LA, Schmid CH, Zhang YL, Castro AF 3rd, Feldman HI, Kusek JW, Eggers P, Van Lente F, Greene T, Coresh J; CKD-EPI (Chronic Kidney Disease Epidemiology Collaboration). A new equation to estimate glomerular filtration rate. *Ann Intern Med*. 2009;150(9):604-12.

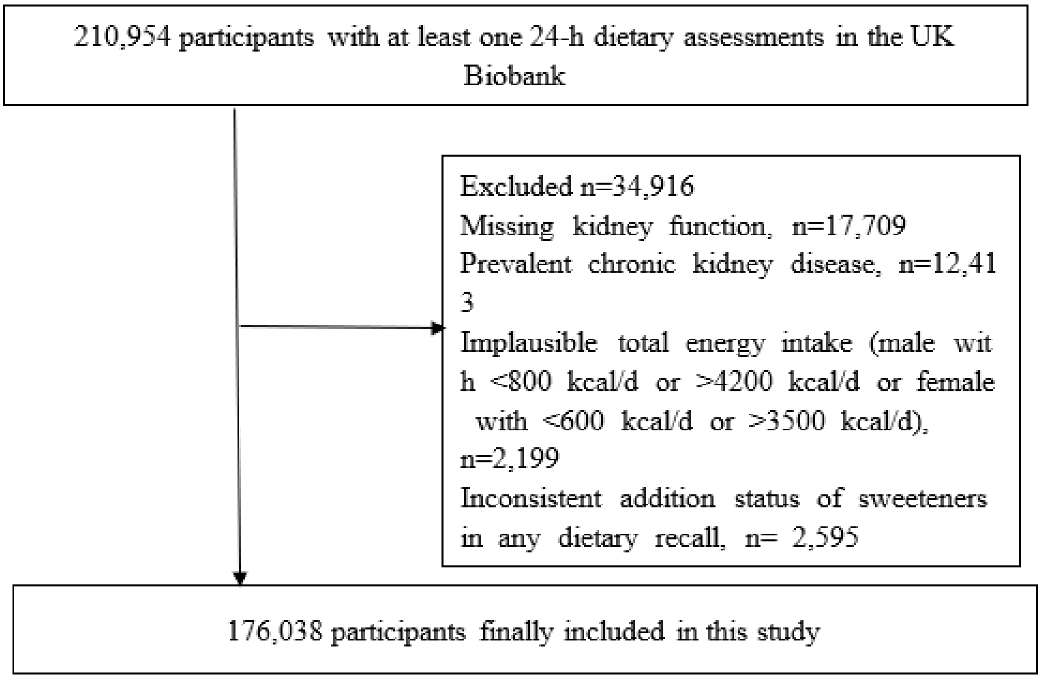

Figure S1. Flow chart of the participants in the UK biobank study.

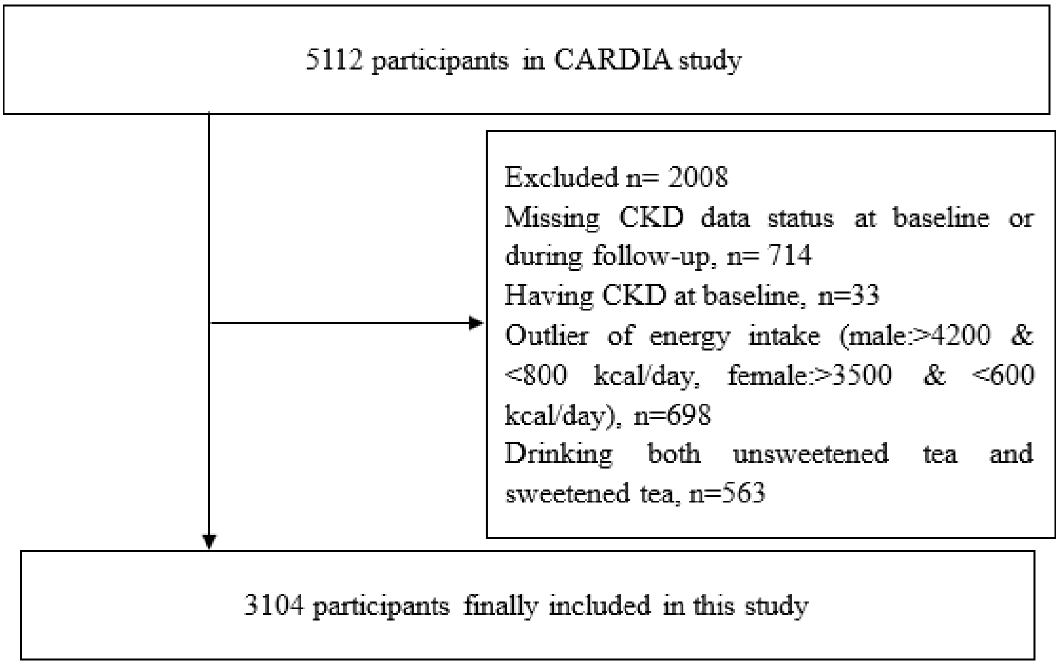

Figure S2. Flow chart of the participants in the CARDIA study.

Table S1. Chronic kidney disease definitions used in the UK Biobank study.

Disease	ICD-9	ICD-10	OPCS-4
Chronic kidney disease	585,5859	I12.0, I13.1, I13.2, N18.0, N18.3, N18.4, N18.5, N18.8, N18.9	M01

Abbreviations: ICD, International Classification of Diseases; OPCS, the Office of Population Censuses and Surveys Classification of Interventions and Procedures.

Table S2. Hazard ratios (95% CI) of new-onset CKD according to total tea consumption in the UK Biobank.

Total tea	Nonconsumers	Tea Consumers, drinks/d				
		≤1.5	>1.5-2.5	>2.5-3.5	>3.5-4.5	>4.5
Events (rates*)	606(1.7)	446(1.4)	614(1.6)	642(1.6)	557(1.7)	670(1.9)
Adjusted Model 1†	ref	0.77(0.68, 0.87)	0.80(0.72, 0.90)	0.82(0.73, 0.91)	0.87(0.77, 0.97)	0.98(0.88, 1.10)
Adjusted Model 2†	ref	0.87(0.77, 0.99)	0.89(0.79, 1.00)	0.85(0.75, 0.95)	0.85(0.75, 0.96)	0.88(0.77, 0.99)

*Incidence rates per 1000 person years.

† Model 1: Adjusted for age, sex, and ethnicities;

Model 2: adjusted for the covariates in Model 1 and further adjusted for educational attainment, smoking status, alcohol consumption, physical activity, total energy intake, coffee consumption, body mass index, systolic blood pressure, diastolic blood pressure, glucose, triglycerides, total cholesterol, high-density lipoprotein cholesterol, estimated glomerular filtration rate and urine albumin-to-creatinine ratio.

Table S3. Hazard ratios (95% CI) of new-onset CKD according to tea consumption stratified by added milk and sweeteners in tea in the UK Biobank*.

Tea consumption, drinks/d	Total	No of events	Adjusted Model 1†		Adjusted Model 2†	
			HR (95%CI)	*P value*	HR (95%CI)	*P value*
**Nonconsumers**	29127	606	ref		ref	
**Tea consumers**						
**Not adding milk or sweeteners**						
≤1.5	7189	120	0.74(0.61, 0.90)	0.003	0.88(0.72, 1.07)	0.203
>1.5-2.5	5373	98	0.76(0.61, 0.94)	0.011	0.94(0.75, 1.16)	0.555
>2.5-3.5	4455	77	0.70(0.55, 0.88)	0.003	0.84(0.66, 1.07)	0.162
>3.5-4.5	3180	58	0.75(0.57, 0.98)	0.037	0.83(0.63, 1.09)	0.181
>4.5	3483	67	0.85(0.66, 1.09)	0.198	0.88(0.68, 1.14)	0.327
**Only adding milk**						
≤1.5	11542	192	0.73(0.62, 0.86)	<0.001	0.86(0.73, 1.02)	0.082
>1.5-2.5	18131	324	0.74(0.65, 0.85)	<0.001	0.84(0.73, 0.96)	0.012
>2.5-3.5	20201	364	0.76(0.66, 0.86)	<0.001	0.80(0.69, 0.91)	0.001
>3.5-4.5	17548	329	0.79(0.69, 0.90)	0.001	0.80(0.69, 0.92)	0.002
>4.5	19750	401	0.90(0.79, 1.02)	0.094	0.83(0.72, 0.96)	0.010
**Only adding sweeteners**						
≤1.5	895	15	0.69(0.41, 1.15)	0.158	0.72(0.43, 1.20)	0.212
>1.5-2.5	662	16	0.95(0.58, 1.57)	0.853	0.88(0.53, 1.44)	0.600
>2.5-3.5	520	12	0.87(0.49, 1.53)	0.619	0.89(0.50, 1.57)	0.677
>3.5-4.5	316	10	1.19(0.63, 2.22)	0.593	1.01(0.54, 1.89)	0.975
>4.5	385	17	1.82(1.13, 2.95)	0.015	1.71(1.05, 2.78)	0.031
**Adding both milk and sweeteners**						
≤1.5	4460	100	0.99(0.80, 1.22)	0.900	0.94(0.76, 1.16)	0.572
>1.5-2.5	5564	135	1.03(0.85, 1.24)	0.791	0.96(0.79, 1.16)	0.659
>2.5-3.5	5477	148	1.10(0.92, 1.31)	0.315	0.93(0.77, 1.12)	0.459
>3.5-4.5	4556	136	1.23(1.02, 1.48)	0.031	0.99(0.81, 1.20)	0.892
>4.5	4972	158	1.29(1.08, 1.54)	0.005	0.95(0.79, 1.15)	0.606

* 8252 participants with inconsistent addition status of milk in any dietary recall were excluded.

†Adjusted Model 1: Adjusted for age, sex, and ethnicities;

Adjusted Model 2: adjusted for the covariates in Model 1 and further adjusted for educational attainment, smoking status, alcohol consumption, physical activity, total energy intake, coffee consumption, body mass index, systolic blood pressure, diastolic blood pressure, glucose, triglycerides, total cholesterol, high-density lipoprotein cholesterol, estimated glomerular filtration rate and urine albumin-to-creatinine ratio.

Table S4. Sensitivity analysis for hazard ratios (95% CI) of new-onset CKD according to tea consumption in the UK Biobank.

	Nonconsumers	Tea Consumers, drinks/d				
		≤1.5	>1.5-2.5	>2.5-3.5	>3.5-4.5	>4.5
**Sensitivity analyses 1: Excluding participants who developed CKD in the first two years of follow-up**						
**Unsweetened tea**						
Events (rates*)	595(1.7)	319(1.3)	454(1.5)	458(1.4)	398(1.5)	476(1.6)
Adjusted HR (95% CI)†	ref	0.86(0.75, 0.98)	0.89(0.78, 1.01)	0.82(0.72, 0.93)	0.82(0.71, 0.95)	0.86(0.75, 0.99)
**Sweetened tea**						
Events (rates*)	595(1.7)	116(1.8)	149(1.9)	166(2.3)	141(2.4)	179(2.8)
Adjusted HR (95% CI)†	ref	0.92(0.75, 1.12)	0.92(0.77, 1.11)	0.97(0.81, 1.17)	0.96(0.78, 1.17)	1.02(0.84, 1.25)
**Sensitivity analyses 2: Excluding participants with missing covariates**						
**Unsweetened tea**						
Events (rates*)	495(1.7)	287(1.3)	400(1.5)	381(1.4)	332(1.4)	399(1.6)
Adjusted HR (95% CI)†	ref	0.89(0.77, 1.03)	0.89(0.77, 1.02)	0.78(0.68, 0.90)	0.79(0.68, 0.92)	0.85(0.73, 0.99)
**Sweetened tea**						
Events (rates*)	495(1.7)	96(1.7)	129(2.0)	138(2.2)	120(2.4)	148(2.7)
Adjusted HR (95% CI)†	ref	0.90(0.72, 1.13)	0.99(0.81, 1.21)	0.97(0.80, 1.19)	0.97(0.78, 1.20)	1.04(0.84, 1.29)
**Sensitivity analyses 3: Further adjustments for consumption of water, low/non sugar sugar-sweetened beverages, and sugar-sweetened beverages**						
**Unsweetened tea**						
Events (rates*)	606(1.7)	327(1.3)	461(1.5)	474(1.5)	409(1.5)	488(1.7)
Adjusted HR (95% CI)†	ref	0.86(0.75, 0.99)	0.89(0.79, 1.02)	0.85(0.74, 0.97)	0.85(0.74, 0.98)	0.90(0.78, 1.04)
**Sweetened tea**						
Events (rates*)	606(1.7)	119(1.8)	153(2.0)	168(2.3)	148(2.5)	182(2.8)
Adjusted HR (95% CI)†	ref	0.91(0.74, 1.11)	0.92(0.76, 1.11)	0.96(0.80, 1.16)	0.99(0.81, 1.21)	1.04(0.85, 1.27)

**Sensitivity analyses 4: Further considering the type of sweeteners added into tea**						
**Sweetened tea adding sugar**						
Events (rates*)	606(1.7)	60(1.4)	79(1.6)	95(2.0)	88(2.3)	99(2.4)
Adjusted HR (95% CI)†	ref	0.80(0.61, 1.05)	0.84(0.66, 1.08)	0.97(0.77, 1.23)	1.01(0.79, 1.29)	0.90(0.71, 1.15)
**Sweetened tea adding sugar artificial sweeteners**						
Events (rates*)	606(1.7)	54(2.5)	69(2.7)	76(3.0)	69(3.2)	84(3.4)
Adjusted HR (95% CI)†	ref	1.04(0.79, 1.38)	1.01(0.79, 1.31)	1.05(0.82, 1.35)	1.16(0.89, 1.51)	1.25(0.97, 1.61)

*Incidence rates per 1000 person years.

† Results were adjusted for age, sex, ethnicities, educational attainment, smoking status, alcohol consumption, physical activity, total energy intake, coffee consumption, body mass index, systolic blood pressure, diastolic blood pressure, glucose, triglycerides, total cholesterol, high-density lipoprotein cholesterol, estimated glomerular filtration rate and urine albumin-to-creatinine ratio.

Table S5. Baseline population characteristics according to tea consumption categories in the CARDIA study*.

	Nonconsumers	Tea Consumers		*P* value
		Unsweetened	Sweetened	
**N**	871	1972	261	
**Age, years**	24.8 (3.7)	25.4 (3.4)	24.3 (3.8)	< 0.001
**Male, No. (%)**	420 (48.2)	749 (38.0)	118 (45.2)	< 0.001
**White, No. (%)**	385 (44.2)	1199 (60.8)	87 (33.3)	< 0.001
**Education, years**	13.3 (1.8)	14.1 (1.8)	13 (1.7)	< 0.001
**Smoking status, No. (%)**				< 0.001
Never	462 (53.4)	1199 (61.1)	166 (64.1)	
Former	99 (11.4)	305 (15.5)	21 (8.1)	
Current	304 (35.1)	458 (23.3)	72 (27.8)	
**Alcohol consumption, drinks/week**	4.8 (8.1)	4.1 (6.6)	3.8 (6.3)	0.022
**Physical activity, exercise units**	370 (268.6)	422.4 (283.2)	349.3 (293.7)	< 0.001
**Energy intake, kcal/d**	2437.3 (754.1)	2360.5 (712.3)	2411.7 (778.0)	0.030
**Coffee consumption, cups/d**	1 (1.5)	1.2 (1.4)	0.8 (1.4)	< 0.001
**Biological factors**				
Body mass index, kg/m^2	25.0 (5.0)	24.2 (4.6)	25.1 (5.3)	< 0.001
Systolic blood pressure, mmHg	111.6 (11.2)	109.7 (10.7)	110.4 (10.7)	< 0.001
Diastolic blood pressure, mmHg	69.5 (9.8)	68.6 (9.0)	68.4 (10.5)	0.051
Glucose, mg/dL	83.0 (14.4)	82.1 (11.5)	81.1 (9.3)	0.051
Triglycerides, mg/dL	73.2 (48.0)	71.9 (47.2)	74.3 (44.3)	0.644
Total cholesterol, mg/dL	179.7 (33.8)	177.9 (32.9)	175.4 (32.1)	0.161
High-density lipoprotein cholesterol, mg/dL	52.8 (13.8)	54.2 (12.9)	51.2 (12.9)	< 0.001
Estimated glomerular filtration rate, mL/min/1.73 m^2	96.8 (17.2)	94.9 (17.3)	97.8 (17.5)	0.004

*Values are presented as means (SD) or proportions.

Table S6. Hazard ratios (95% CI) of new-onset CKD according to unsweetened tea consumption in various subgroups in the UK Biobank.

	Non-consumers	Tea Consumers, drinks/d					P for interaction
		≤1.5	>1.5-2.5	>2.5-3.5	>3.5-4.5	>4.5	
**Age, years**							0.643
<60	ref	0.73(0.56,0.96)	0.88(0.70,1.11)	0.85(0.67,1.07)	0.76(0.59,0.98)	0.84(0.67,1.07)	
≥60	ref	0.94(0.80,1.10)	0.92(0.79,1.06)	0.86(0.74,1.00)	0.89(0.76,1.04)	0.91(0.77,1.06)	
**Sex**							0.192
Female	ref	0.82(0.67,0.99)	0.79(0.66,0.95)	0.72(0.60,0.86)	0.75(0.62,0.90)	0.83(0.69,0.99)	
Male	ref	0.91(0.75,1.10)	0.98(0.83,1.17)	0.96(0.80,1.14)	0.92(0.76,1.11)	0.88(0.73,1.07)	
**Body mass index, kg/m^2**							0.688
<30	ref	0.85(0.72,1.02)	0.93(0.79,1.08)	0.82(0.70,0.96)	0.82(0.69,0.97)	0.86(0.73,1.01)	
≥30	ref	0.85(0.68,1.06)	0.76(0.61,0.94)	0.81(0.66,1.00)	0.81(0.65,1.00)	0.83(0.67,1.02)	
**Smoking status**							0.700
Never	ref	0.79(0.65,0.96)	0.81(0.68,0.97)	0.76(0.64,0.91)	0.81(0.67,0.97)	0.84(0.70,1.00)	
Ever	ref	0.93(0.77,1.12)	0.95(0.80,1.13)	0.88(0.74,1.05)	0.83(0.69,1.01)	0.89(0.74,1.07)	
**Alcohol consumption, times/week**							0.840
**<1**	ref	0.78(0.61,1.00)	0.84(0.67,1.05)	0.83(0.67,1.02)	0.76(0.61,0.95)	0.81(0.66,0.99)	
≥1	ref	0.90(0.76,1.06)	0.91(0.78,1.07)	0.84(0.72,0.99)	0.88(0.74,1.04)	0.91(0.77,1.08)	
**Optimal physical activity**							0.591
No	ref	0.86(0.70,1.06)	0.93(0.77,1.13)	0.87(0.72,1.05)	0.82(0.67,1.01)	0.96(0.78,1.16)	
Yes	ref	0.84(0.70,1.01)	0.83(0.70,0.99)	0.76(0.64,0.90)	0.79(0.66,0.95)	0.77(0.64,0.92)	
**Energy intake**							0.804
< Median	ref	0.90(0.75,1.08)	0.84(0.71,1.00)	0.82(0.69,0.97)	0.79(0.66,0.95)	0.84(0.70,1.01)	

≥ Median	ref	0.82(0.66,1.00)	0.93(0.78,1.12)	0.84(0.70,1.01)	0.85(0.71,1.03)	0.87(0.72,1.05)

*Adjusted, if not stratified, for age, sex, ethnicities, educational attainment, smoking status, alcohol consumption, physical activity, total energy intake, coffee consumption, body mass index, systolic blood pressure, diastolic blood pressure, glucose, triglycerides, total cholesterol, high-density lipoprotein cholesterol, estimated glomerular filtration rate and urine albumin-to-creatinine ratio.
